# Supplementary material for: Screening plans for SARS-CoV-2 based on sampling and rotation: An example in a European school setting
Source: PLoS One. 2021 Sep 10;16(9):e0257099. doi: 10.1371/journal.pone.0257099 (PMC8432749; doi:10.1371/journal.pone.0257099)
Supplement: S1 Table — Mean and 90th percentile of the number of lost infection-days by screening plan (A1, A2, B1, B2, C, D), under different epidemic scenarios (R0 and T), assuming that individual tests have sensitivity 0.7 and maximum specificity. (PDF) [file pone.0257099.s001.pdf]

Scenario			Screening plan											
			A1		A2		B1		B2		C		D	
R_0	T	β	mean	90th p.	mean	90th p.	mean	90th p.	mean	90th p.	mean	90th p.	mean	90th p.
1.1	21	0.05	6.2	13	10.8	24	10.2	22	16.7	38	12.6	32	16.4	37
2.0	21	0.10	6.6	14	12.9	30	11.4	26	21.1	50	18.1	46	20.1	47
3.0	21	0.14	7.3	16	15.6	37	12.7	29	26.4	65	22.9	58	23.3	55
5.0	21	0.24	8.5	20	22.2	59	16.2	38	36.5	93	31.9	80	28.4	68
1.1	14	0.08	6.1	13	10.4	24	9.6	21	15.5	37	12.8	34	14.8	36
2.0	14	0.14	6.7	15	13.5	33	11.2	26	20.7	52	18.8	48	19.0	46
3.0	14	0.21	7.7	17	17.8	45	13.4	31	27.4	71	25.0	63	23.1	56
5.0	14	0.36	9.9	23	28.0	76	17.2	42	41.5	109	34.5	87	30.1	72
1.1	7	0.16	5.5	12	9.5	23	8.3	20	13.2	34	11.6	31	12.1	30
2.0	7	0.29	7.2	17	15.5	43	11.6	29	21.8	59	19.2	52	17.9	46
3.0	7	0.43	9.1	22	23.9	68	15.1	38	32.2	87	27.3	71	23.5	60
5.0	7	0.71	14.4	37	41.5	106	22.5	57	51.0	120	40.8	96	32.8	78

Table S1: Mean and 90th percentile of the number of lost infection-days by screening plan (A1, A2, B1, B2, C, D), under different epidemic scenarios (R_0 and T), assuming that individual tests have sensitivity 0.7 and maximum specificity.
