# Supplementary material for: Screening plans for SARS-CoV-2 based on sampling and rotation: An example in a European school setting
Source: PLoS One. 2021 Sep 10;16(9):e0257099. doi: 10.1371/journal.pone.0257099 (PMC8432749; doi:10.1371/journal.pone.0257099)
Supplement: S2 Table — Mean and 90th percentile of the number of lost infection-days by screening plan (A1, A2, B1, B2, C, D), under different epidemic scenarios (R0 and T), assuming that individual tests have sensitivity 0.9 and maximum specificity. (PDF) [file pone.0257099.s002.pdf]

Scenario			Screening plan											
			A1		A2		B1		B2		C		D	
R_0	T	β	mean	90th p.	mean	90th p.	mean	90th p.	mean	90th p.	mean	90th p.	mean	90th p.
1.1	21	0.05	4.6	9	8.5	18	8.0	16	14.1	32	13.0	30	13.7	30
2.0	21	0.10	4.8	10	9.2	20	8.3	17	15.6	36	14.6	34	14.7	33
3.0	21	0.14	5.5	11	12.3	29	10.0	22	21.1	51	19.6	47	18.1	42
5.0	21	0.24	6.7	15	18.2	46	12.4	29	30.0	78	26.7	66	22.7	54
1.1	14	0.08	4.7	9	8.4	18	7.7	16	13.2	30	12.7	30	12.4	28
2.0	14	0.14	4.9	10	9.4	21	8.2	18	14.9	36	14.5	35	13.9	33
3.0	14	0.21	5.9	13	14.6	37	10.5	24	23.3	59	20.7	52	18.7	45
5.0	14	0.36	7.8	18	24.1	66	14.2	34	37.1	99	29.7	76	23.9	58
1.1	7	0.16	4.6	10	8.4	20	7.0	16	11.7	30	11.0	28	10.6	26
2.0	7	0.29	5.0	11	10.5	27	8.4	20	14.8	39	13.4	35	12.6	31
3.0	7	0.43	7.5	18	21.5	62	12.5	31	29.1	81	23.6	62	20.1	51
5.0	7	0.71	12.0	32	38.6	102	19.1	49	47.4	115	37.0	89	28.2	69

Table S2: Mean and 90th percentile of the number of lost infection-days by screening plan (A1, A2, B1, B2, C, D), under different epidemic scenarios (R_0 and T), assuming that individual tests have sensitivity 0.9 and maximum specificity.
